# Serum transgelin is a novel prognostic biomarker for COVID-19 patients

**DOI:** 10.3389/fimmu.2024.1423182

**Published:** 2024-11-29

**Authors:** Lei Gao, Ying Liu, Qi-Yuan He, Yu Wang, Ya-Lin Jiang, Jin Yang, Lin Fu, Hui Zhao

**Affiliations:** ^1^ Department of Respiratory and Critical Care Medicine, Second Affiliated Hospital of Anhui Medical University, Hefei, Anhui, China; ^2^ Institute of Respiratory Diseases, Second Affiliated Hospital of Anhui Medical University, Hefei, Anhui, China; ^3^ Department of Respiratory and Critical Care Medicine, Bozhou People’s Hospital, Bozhou, Anhui, China

**Keywords:** transgelin, cohort study, COVID-19, severity, prognosis

## Abstract

**Background:**

Transgelin is a central actin-binding protein of the calponin family and involved in the process of multiple pulmonary diseases. Nevertheless, the role of transgelin in Coronavirus disease 2019 (COVID-19) patients is confusing.

**Methods:**

All 317 COVID-19 patients were recruited from two hospital. Peripheral blood was collected from the fasting patients at the onset and convalescent phases. Demographic data and clinical information were obtained. The expression of serum transgelin was estimated using ELISA.

**Results:**

The expression of serum transgelin on admission was gradually elevated in parallel with the increased severity scores of COVID-19. After treatment, serum transgelin expression was reduced during the convalescent phase. Spearman correlative analyses found that serum transgelin expression was closely correlated to lots of clinical parameters. Besides, serum transgelin was positively associated with severity scores. Follow-up research found that serum higher transgelin on admission elevated the risks of mechanical ventilation, vasoactive agent utilization, ICU admission, death, and longer hospital stays during hospitalization through a prospective cohort study. Additionally, there were similarly predictive capacities for critical patients and death between serum transgelin on admission and severity scores among COVID-19 patients.

**Conclusions:**

The expression of serum transgelin is positively with the severity and poorly prognostic outcomes among COVID-19 patients, indicating that transgelin is implicated in the pathological process of COVID-19. Transgelin can assist in the risk stratification and revealing the pathological mechanisms of COVID-19.

## 1 Background

Coronavirus disease 2019 (COVID-19) is an infectious disease evoked by severe acute respiratory syndrome coronavirus 2 (SARS-CoV-2) (1, 2). It occurred at the end of 2019, and the first case was found and reported in Wuhan City of China (3). Due to the high transmissibility rate, COVID-19 has spread quickly to the entire world from China, and led to a pandemic (4). The former data revealed that an estimated 2% cases die for COVID-19, and there are 5~10% COVID-19 patients progressing to acute respiratory distress syndrome (ARDS) during the initial three years of the pandemic (5–7). A majority of COVID-19 patients finally develop into mild or moderate illness (8). Although the mortality rate is decreased because of the subsequent omicron variants, the frequency of the infected patients is elevated and incurs a relative rise of the dead cases (9). Due to the pandemic all over the world, COVID-19 has brought a severe and threatening challenge for medical system and global public health crisis.

Transgelin, also called SM22α, is a central actin-binding protein of the calponin family and in the cytoskeleton (10, 11). The previous investigation found that transgelin is widely and richly expressed in vascular smooth muscle cells. Transgelin expression is regarded as an earlier biomarker of smooth muscle differentiation (12). However, the latest researches hinted that transgelin exerts the important roles in tumor‐suppressive and oncogenic functions in the different types of cancers. The expression of transgelin is reduced in bladder carcinoma and prostate cancer (13, 14). On the contrary, the levels of transgelin are elevated in colorectal cancer and ovarian cancer (15, 16). It is widely known that transgelin takes part in the process of pulmonary diseases. Elevated transgelin can promote the progression of lung cancer (17, 18). In addition, the expression of transgelin is elevated in the airway smooth muscle cells of rodent asthma models (19). Moreover, pulmonary transgelin is up-regulated in pulmonary epithelial cells of mice during bleomycin-evoked lung fibrosis and lung tissues from patients with idiopathic pulmonary fibrosis (20). However, the expression and change of transgelin are unclear in patients with COVID-19.

Up to now, there are no definite evidence and relative report about the role of transgelin in COVID-19 patients. As a consequence, COVID-19 patients were recruited and serum specimens were collected. The level of serum transgelin was measured. The relationships between serum transgelin with the severity and outcomes were analyzed through a perspective cohort study. The current research may provide an important clue about the application which was used as a biomarker to evaluate the severity and predict clinical outcomes, and guide the clinical therapeutics of COVID-19 cases.

## 2 Materials and methods

### 2.1 Study design and human participants

All patients admitted to Department of Respiratory and Critical Care Medicine, Second Affiliated Hospital of Anhui Medical University from December 2022 to May 2023 were enrolled. All cases must be confirmed with SARS-CoV-2 infection through reverse transcription-polymerase chain reaction in pharyngeal swab specimens. At last, 317 inpatients with COVID-19 were enrolled in this research. Clinical characteristics, demographic information, and blood specimens were obtained from all COVID-19 patients. The inclusion criteria were: (1) Age was more than 18 years; (2) All subjects were newly diagnosed and confirmed with SARS-CoV-2 by RT-PCR; 3 The onset time was shorter than 48 hours; (3) All subjects were volunteered to participate this research. The exclusion criteria were: (1) Pregnant women; (2) Patients were accompanied with malignant tumors, autoimmune diseases, and respiratory diseases; (3) Antibiotics and antiviral drugs were taken in one week before this admission. After admission, the severity was estimated by the scoring system, including SMART-COP, CURB-65, CURXO, COVID-GRAM, Pneumonia Severity Index (PSI), Coronavirus Clinical Characterization Consortium Mortality (4C)-Mortality, Diagnosis and Treatment Protocol for Novel Coronavirus Pneumonia (DTPNCP), MuLBSTA, and A-DROP (21–27).

### 2.2 Enzyme-linked immunosorbent assay

Blood specimens were centrifuged. Human transgelin ELISA kits (CSB-E17844h) were purchased from Cusabio, Wuhan, China (https://www.cusabio.com/). Then, the expression of serum transgelin was detected in accordance with the published methods in detail (28, 29).

### 2.3 Statistical analysis

Continuous variables were shown as mean or median. The categorical variables were represented as number (percentage). The difference of basic data was compared and evaluated by the Fisher test, one-way analysis of variance (ANOVA), or non-parametric test. The relationships between serum transgelin and clinical characteristics were estimated via Spearman correlation analyses. Additionally, the relationships between serum transgelin and severity scores were estimated by linear and logistic regression analyses. The correlations between serum transgelin and outcomes were assessed through logistic regression analyses and χ^2^ test or Fisher exact probability. The predictive powers for critical cases and death were appraised through receiver operating characteristic (ROC) curve. The comparison of serum transgelin levels between onset and convalescent was assessed by paired Student’s t-test. A *P*<0.05 was statistical significance.

## 3 Results

### 3.1 Basic information of COVID-19 patients

COVID-19 patients were divided into T1 group (the expression of serum transgelin was lower than 0.06 ng/mL), T2 group (the expression of serum transgelin was from 0.06 to 1.02 ng/mL), and T3 group (the expression of serum transgelin was higher than 1.02 ng/mL) in accordance with the tertitles of transgelin expression. Then, clinical and demographic characteristics were analyzed and compared. As represented in **Table 1**, the average age and the number of males were highest in the T3 group. The comorbidities were analyzed. There was no difference of hypertension, diabetes mellitus, coronary heart diseases, chronic liver diseases, hepatitis B, and chronic kidney diseases among COVID-19 patients with different subgroups. However, the numbers of other chronic heart disease and cerebrovascular diseases in T3 group were more than T1 and T2 groups (**Table 1**). In addition, heart rate, the number of corticosteroids therapy, anticoagulant therapy, white blood cell (WBC), neutrophil, alanine aminotransferase (ALT), aspartate aminotransferase (AST), urea nitrogen, D-Dimer, C-reactive protein (CRP), and procalcitonin (PCT) were gradually elevated in parallel with serum transgelin expression (**Table 1**). On the contrary, the levels of partial pressure of oxygen (PaO_2_), fraction of inspiration O_2_ (FiO_2_), oxygen saturation (SpO2), and PaO2/FiO_2_ were reduced with the increased serum transgelin (**Table 1**).

**Table 1:** Demographic characteristics and clinical information at baseline.

Characteristic	Tertile of serum transgelin (ng/mL)			*P*
	T1 (≤0.60)	T2 (0.60~1.02)	T3 (≥1.02)	
N	106	105	106	
Age, years	67.7±16.0	68.9±14.0	73.7±13.2	**0.006**
Male, n (%)	57 (53.8)	53 (50.5)	74 (69.8)	**0.010**
BMI	24.3±4.7	24.7±4.4	24.7±3.93	0.722
Smoker, n (%)	15 (14.2)	11 (10.5)	17 (16.0)	0.517
Systolic pressure (mmHg)	132.2±21.2	137.7±19.2	133.4±21.6	0.127
Diastolic pressure (mmHg)	77.2±11.7	81.2±11.3	79.0±13.8	0.059
Hypertension, n (%)	51 (48.1)	49 (46.7)	53 (50.0)	0.889
Diabetes mellitus, n (%)	22 (20.8)	20 (19.0)	30 (28.3)	0.232
Coronary heart diseases, n (%)	23 (21.7)	19 (18.1)	19 (17.9)	0.742
Other chronic heart disease, n (%)	5 (4.7)	8 (7.6)	18 (17.0)	**0.009**
Cerebrovascular diseases, n (%)	26 (24.6)	14 (13.3)	28 (26.4)	**0.040**
Chronic liver diseases, n (%)	4 (3.8)	4 (3.8)	3 (2.8)	1.000
Hepatitis B, n (%)	3 (2.8)	3 (2.9)	2 (1.9)	1.000
Chronic kidney diseases, n (%)	9 (8.5)	9 (8.6)	9 (8.5)	1.000
Heart rate (Breath/minute)	90.2±10.6	87.4±16.3	93.5±10.5	**0.042**
Respiratory rate (Breath/minute)	20.8±9.3	20.4±4.0	22.2±8.6	0.205
Temperature (°C)	36.7±1.0	36.8±0.8	36.6±0.6	0.475
PaO_2_ (mm Hg)	88.3 (73.0, 103.3)	86.1 (68.2, 104.0)	73.1 (59.8, 91.9)	**0.001**
FiO_2_ (%)	29.9±4.2	30.3±4.6	35.3±3.5	**<0.001**
SpO_2_ (%)	95.8±2.7	94.3±5.1	91.1±6.7	**<0.001**
PaO2/FiO_2_ (%)	307.1 (260.7, 355.2)	296.6 (230.5, 354.5)	237.4 (162.5, 312.6)	**<0.001**
Corticosteroids therapy, n (%)	80 (75.5)	82 (78.1)	95 (89.6)	**0.016**
Antibiotics therapy, n (%)	101 (95.3)	99 (94.3)	104 (98.1)	0.363
Antiviral therapy, n (%)	37 (34.9)	47 (44.8)	54 (50.9)	0.061
Anticoagulant therapy, n (%)	40 (37.7)	63 (60.0)	76 (71.7)	**<0.001**
WBC (10^9^/L)	6.6 (5.1, 8.5)	7.2 (5.1, 9.7)	8.6 (5.5, 12.0)	**0.005**
Neutrophil (10^9^/L)	4.6 (3.5, 6.5)	5.4 (3.6, 7.8)	6.6 (3.7, 9.6)	**0.002**
Lymphocyte (10^9^/L)	1.1 (0.6, 1.4)	1.0 (0.7, 1.5)	0.8 (0.5, 1.5)	0.241
Monocyte (10^9^/L)	0.5 (0.4, 0.6)	0.5 (0.3, 0.6)	0.5 (0.3, 0.7)	0.763
ALT (U/L)	22.0 (15.0, 38.5)	22.0 (18.0, 36.7)	29.0 (19.0, 48.5)	**0.011**
AST (U/L)	26.0 (20.0, 37.0)	26.0 (19.8, 37.0)	32.0 (20.0, 49.0)	**0.026**
Uric acid (μmol/L)	267.0 (195.8, 351.6)	244.2 (201.7, 312.1)	230.9 (178.8, 293.0)	0.244
Urea nitrogen (mmol/L)	6.3 (4.7, 8.1)	6.2 (4.7, 8.0)	7.1 (5.4, 10.6)	**0.012**
Creatinine (μmol/L)	65.5 (56.3, 85.0)	66.0 (53.6, 83.1)	67.5 (56.0, 83.0)	0.944
CK (U/L)	56.0 (36.0, 79.0)	47.0 (28.5, 73.0)	55.0 (35.0, 112.5)	0.087
CK-MB (U/L)	12.5 (7.0, 18.0)	12.0 (7.0, 17.0)	14.0 (7.0, 24.0)	0.142
Myoglobin (ng/mL)	51.3 (34.1, 73.0)	94.4 (27.9, 240.1)	81.2 (40.2, 296.1)	0.108
D-Dimer (ng/mL)	0.6 (0.4, 1.1)	0.5 (0.3, 1.6)	1.0 (0.4, 3.0)	**0.003**
CRP (mg/L)	20.1 (5.2, 47.8)	23.6 (5.5, 70.8)	29.0 (10.8, 98.9)	**0.046**
IL-6 (pg/mL)	19.0 (5.9, 53.9)	16.4 (5.5, 41.7)	33.3 (8.0, 77.9)	0.128
PCT (ng/mL)	0.05 (0.03, 0.2)	0.05 (0.03, 0.09)	0.07 (0.03, 0.4)	**0.031**
LDH (U/L)	234.0 (184.0, 281.0)	234.0 (192.5, 298.0)	252.0 (183.8, 399.5)	0.066

Bold values indicate statistical significance. BMI, body mass index; PaO_2_, partial pressure of oxygen; FiO_2_, fraction of inspiration O_2_; SpO_2_, oxygen saturation; WBC, white blood cell; ALT, alanine aminotransferase; AST, aspartate aminotransferase; CK, creatine kinase; CK-MB, creatine kinase isoenzyme; CRP, C-reactive protein; IL-6, interleukin-6; PCT, procalcitonin; LDH, lactate dehydrogenase.

### 3.2 The expression of serum transgelin in COVID-19 patients with different severity

**Figure 1:**
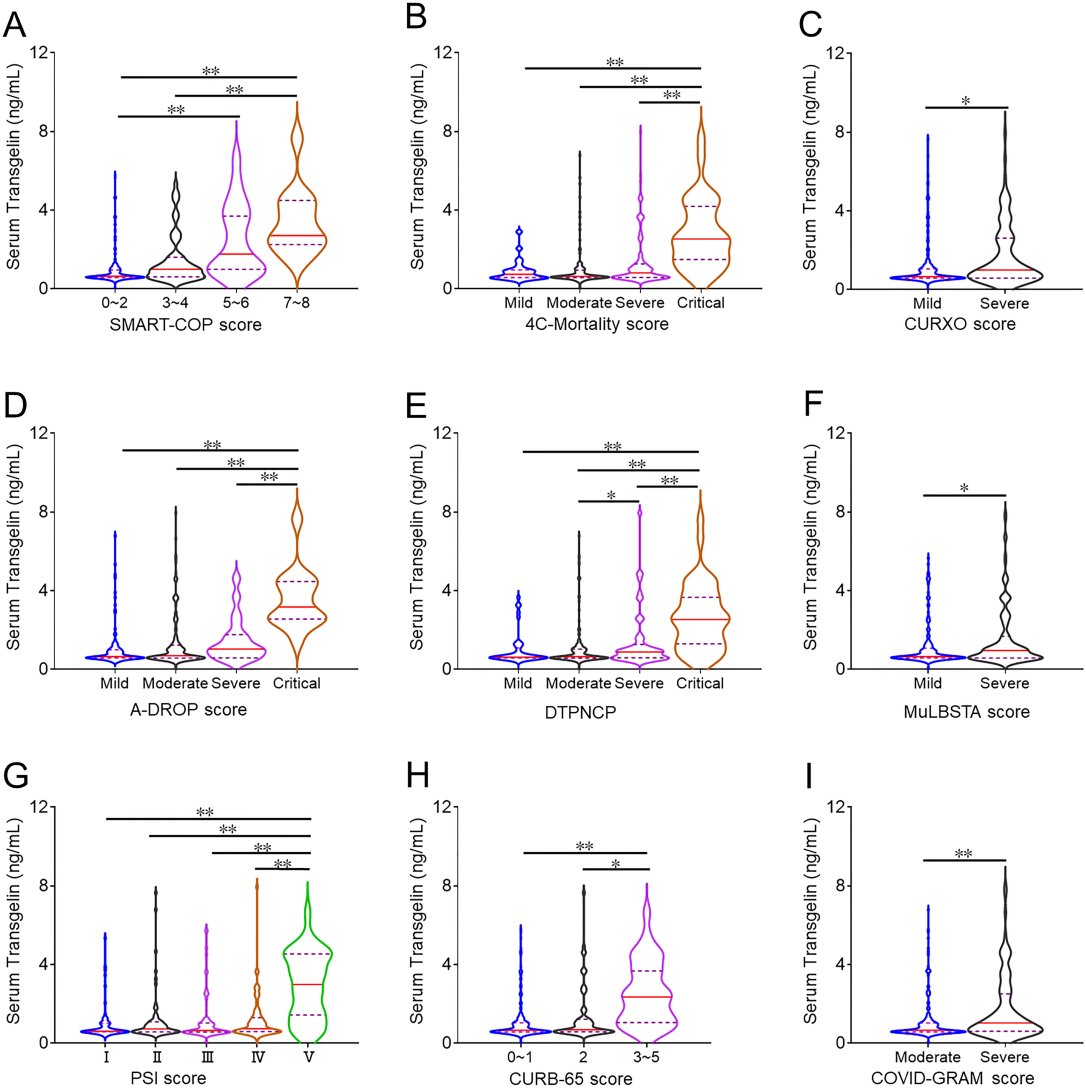
The expression of serum transgelin in COVID-19 patients with different severity scores. The expression of serum transgelin was measured using ELISA. The difference of serum transgelin was compared in COVID-19 patients with different severity scores. **(A)** SMART-COP score. **(B)** 4C-Mortality score. **(C)** CURXO score. **(D)** A-DROP score. **(E)** DTPNCP score. **(F)** MuLBSTA score. **(G)** PSI score. **(H)** CURB-65 score. **(I)** COVID-GRAM score. *P<0.05, **P<0.01.

As shown in **Figure 1A**, the expression of serum transgelin was higher in 5~6 and 7~8 than those in 0~2 and 3~4 scores of SMART-COP. According to 4C-Mortality score, the level of serum transgelin was the highest in critical patients (**Figure 1B**). Moreover, in the basis of CURXO score, serum transgelin expression was obviously upregulated in the severe COVID-19 patients (**Figure 1C**). Based on A-DROP score, serum transgelin expression was the highest in critical COVID-19 cases (**Figure 1D**). In the light of DTPNCP, serum transgelin was increased in severe and critical cases (**Figure 1E**). In addition, we found that serum transgelin was the highest in the highest scores of MuLBSTA, PSI, CURB-65, and COVID-GRAM among COVID-19 patients (**Figures 1F–I**).

### 3.3 The change of serum transgelin in COVID-19 cases between onset and convalescent periods

**Figure 2:**
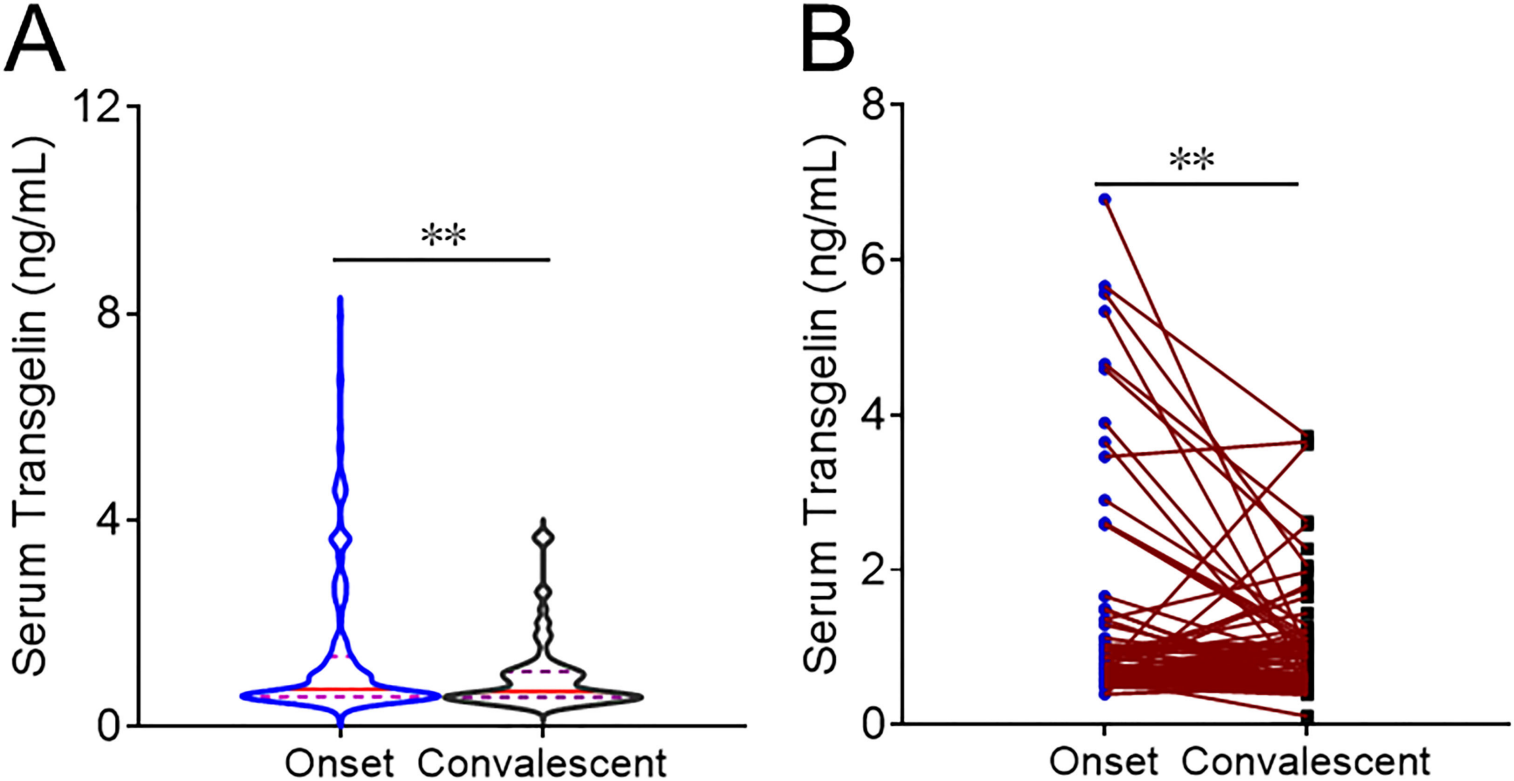
The expression of serum transgelin in COVID-19 patients between onset and convalescent periods. The expression of serum transgelin was detected and compared in COVID-19 cases with different periods. **(A)** The expression of serum transgelin was analyzed in COVID-19 cases at the onset and convalescent phases. **(B)** The expression of serum transgelin was analyzed in the same COVID-19 cases between onset and convalescent periods. **P<0.01.

The level of serum transgelin was compared at the onset and convalescent phases. As shown in **Figure 2A**, serum transgelin was dramatically lower in the convalescent period than those at the onset phase. Moreover, serum transgelin expression was further analyzed in the same subjects between onset and convalescent periods. Serum transgelin expression was substantially decreased in the same COVID-19 patients during the convalescent phase after treatment (**Figure 2B**).

### 3.4 The associations of serum transgelin with basic characteristics of COVID-19 cases

**Figure 3:**
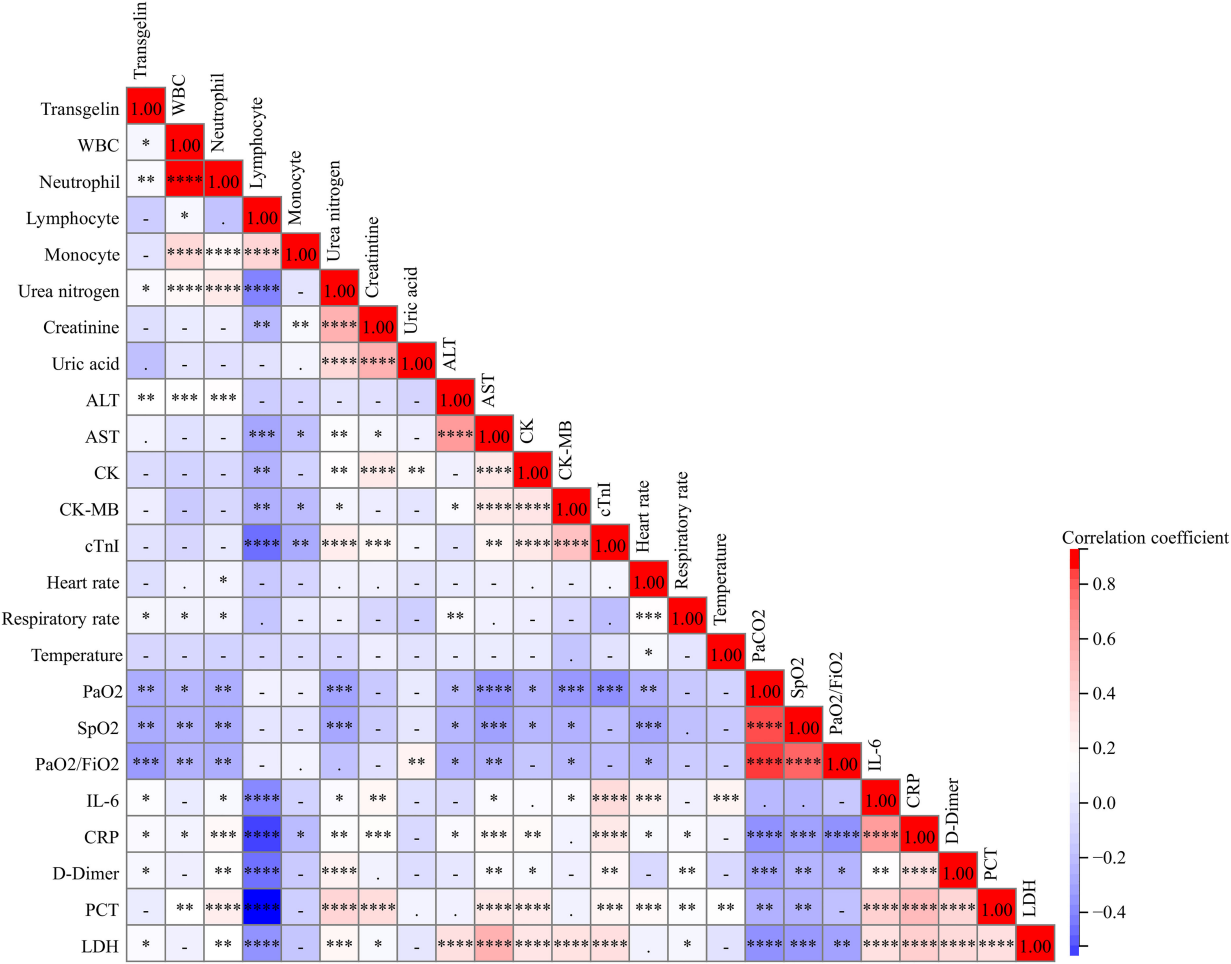
The relationships of serum transgelin with clinical characteristics in COVID-19 patients. The relationships between serum transgelin and clinical characteristics were evaluated among COVID-19 patients by Spearman correlation coefficient or Pearson rank correlation analyses. All characteristics consisted of WBC, neutrophil, lymphocyte, monocyte, urea nitrogen, creatinine, uric acid, ALT, AST, CK, CK-MB, cTnI, heart rate, respiratory rate, temperature, PaO2, SpO2, PaO2/FiO2, IL-6, CRP, D-Dimer, PCT, and LDH. *P<0.05, **P<0.01, ***P<0.001, ****P<0.0001.

The correlations between serum transgelin and clinical parameters were evaluated. Spearman correlative analyses suggested serum transgelin was weakly and positively linked with WBC, neutrophil, urea nitrogen, and ALT (**Figure 3**). Furthermore, there were inverse relationships of serum transgelin with PaO_2_, SpO_2_, and PaO2/FiO_2_ among COVID-19 patients. Besides, positive associations between serum transgelin with interleukin-6 (IL-6), CRP, D-Dimer, and lactate dehydrogenase (LDH) were observed among COVID-19 patients (**Figure 3**).

### 3.5 The associations of serum transgelin with severity of COVID-19 cases

In the univariate linear regression analysis, serum transgelin was positively associated with the scores of SMART-COP (β=0.566), CURB-65 (β=0.264), CURXO (β=0.077), COVID-GRAM (β=5.562), MuLBSTA (β=0.578), A-DROP (β=0.266), PSI (β=9.531), and 4C-Mortality (β=1.021) (**Table 2**). Moreover, univariate logistic regression analysis revealed that serum higher transgelin in T3 subgroup was strongly correlated with the elevated scores of SMART-COP (OR=7.073), CURB-65 (OR=3.042), COVID-GRAM (OR=3.274), MuLBSTA (OR=3.527), A-DROP (OR=4.485), PSI (OR=4.512), 4C-Mortality (OR=7.647), and DTPNCP (OR=4.048) (**Table 2**). In addition, confounding factors were eliminated, multivariate linear and logistic regression analyses also demonstrated that serum transgelin expression was positively related to the severity scores (**Table 2**).

**Table 2:** Associations between serum transgelin and severity in COVID-19 patients.

Models	Variables	Estimated changes by continues transgelin (ng/mL)	Estimated changes (95% CI) by tertiles of transgelin (ng/mL)			*P* trend
			T1 (≤0.60)	T2 (0.60~1.02)	T3 (≥1.02)	
	N	317	106	105	106	
Unadjusted						
	SMART-COP	**0.566 (0.434, 0.699)**	1.0 (Ref)	1.391 (0.560, 3.454)	**7.073 (3.223, 15.523)**	<**0.001**
	CURB-65	**0.264 (0.183, 0.346)**	1.0 (Ref)	1.388 (0.773, 2.629)	**3.042 (1.659, 5.575)**	**0.021**
	CURXO (Severe)	**0.077 (0.039, 0.115)**	1.0 (Ref)	0.964 (0.521, 1.783)	**2.487 (1.398, 4.425)**	0.051
	COVID-GRAM	**5.562 (1.346, 9.778)**	1.0 (Ref)	1.339 (0.651, 2.756)	**3.274 (1.688, 6.349)**	**0.011**
	MuLBSTA	**0.578 (0.234, 0.922)**	1.0 (Ref)	1.788 (0.843, 3.794)	**3.527 (1.738, 7.156)**	**0.001**
	A-DROP	**0.266 (0.181, 0.352)**	1.0 (Ref)	1.452 (0.686, 3.075)	**4.485 (2.266, 8.880)**	**0.001**
	PSI	**9.531 (6.237, 12.825)**	1.0 (Ref)	1.101 (0.490, 2.470)	**4.512 (2.241, 9.084)**	**0.021**
	4C Mortality	**1.021 (0.712, 1.331)**	1.0 (Ref)	2.121 (0.935, 4.811)	**7.647 (3.592, 16.281)**	**0.033**
	DTPNCP	0.229 (0.162, 0.296)	1.0 (Ref)	1.401 (0.733, 2.678)	**4.048 (2.198, 7.455)**	<**0.001**
Adjusted						
	SMART-COP	**0.489 (0.356, 0.622)**	1.0 (Ref)	1.416 (0.544, 3.688)	**5.664 (2.464, 13.017)**	<**0.001**
	CURB-65	**0.178 (0.106, 0.249)**	1.0 (Ref)	1.815 (0.865, 3.808)	**2.663 (1.320, 5.372)**	**0.006**
	CURXO (Severe)	**0.048 (0.011, 0.085)**	1.0 (Ref)	0.979 (0.495, 1.936)	**1.980 (1.042, 3.763)**	0.063
	COVID-GRAM	3.033 (-0.806, 6.873)	1.0 (Ref)	1.789 (0.799, 4.005)	**3.134 (1.485, 6.612)**	**0.002**
	MuLBSTA	**0.373 (0.048, 0.698)**	1.0 (Ref)	2.177 (0.971, 4.883)	**3.189 (1.487, 6.840)**	**0.002**
	A-DROP	**0.176 (0.102, 0.251)**	1.0 (Ref)	2.059 (0.844, 5.022)	**4.301 (1.914, 9.666)**	**0.002**
	PSI	**6.633 (3.634, 9.632)**	1.0 (Ref)	1.308 (0.527, 3.245)	**4.161 (1.873, 9.246)**	**0.013**
	4C Mortality	**0.543 (0.343, 0.744)**	1.0 (Ref)	**3.215 (1.173, 8.810)**	**8.889 (3.500, 22.573)**	<**0.001**
	DTPNCP	0.198 (0.130, 0.266)	1.0 (Ref)	1.509 (0.762, 2.989)	**3.600 (1.885, 6.875)**	**0.021**

Models were adjusted for age, sex, hypertension, diabetes mellitus, coronary heart diseases, other chronic heart disease, cerebrovascular diseases, chronic liver diseases, hepatitis B, and chronic kidney diseases. Bold values indicate statistical significance.

### 3.6 The associations of serum transgelin with prognosis of COVID-19 cases

As shown in **Table 3**, the numbers of mechanical ventilation, the usage of vasoactive agent, ICU admission, death, and longer hospital stays were gradually elevated in parallel with serum transgelin expression. In addition, compared with the T1 subgroup, multivariate logistic regression analysis indicated that the risks of mechanical ventilation (OR=12.570), vasoactive agent usage (OR=20.992), ICU admission (OR=9.857), death (OR=12.635), and longer hospital stays (OR=2.673) were obviously ascended in T3 subgroup (**Table 3**).

**Table 3:** Associations between serum transgelin and prognostic outcomes in COVID-19 patients.

Variables	Serum transgelin (ng/mL)			*P*trend
	T1 (≤0.60)	T2 (0.60~1.02)	T3 (≥1.02)	
N	106	105	106	
Mechanical ventilation				
N, (%)	1 (0.9)	2 (1.9)	17 (16.0)	**<0.001**
Unadjusted RR (95% CI)	Ref (1.0)	2.039 (0.182, 22.832)	**20.056 (2.617, 153.699)**	**0.002**
Adjusted RR (95% CI)	Ref (1.0)	2.107 (0.163, 27.170)	**12.570 (1.326, 119.185)**	**0.036**
Vasoactive agent				
N, (%)	1 (0.9)	6 (5.7)	13 (12.3)	**0.002**
Unadjusted RR (95% CI)	Ref (1.0)	6.364 (0.753, 53.803)	**14.677 (1.884, 114.356)**	**0.001**
Adjusted RR (95% CI)	Ref (1.0)	**16.693 (1.118, 249.158)**	**20.992 (1.409, 312.760)**	**0.015**
ICU admission				
N, (%)	2 (1.9)	4 (3.8)	20 (18.9)	**<0.001**
Unadjusted RR (95% CI)	Ref (1.0)	2.059 (0.369, 11.493)	**12.093 (2.749, 53.196)**	**0.004**
Adjusted RR (95% CI)	Ref (1.0)	2.296 (0.331, 15.943)	**9.857 (1.623, 59.860)**	**0.020**
Death				
N, (%)	2	2 (1.0)	19 (20.8)	**<0.001**
Unadjusted RR (95% CI)	Ref (1.0)	1.827 (0.965, 10.365)	**14.977 (6.573, 137.693)**	**<0.001**
Adjusted RR (95% CI)	Ref (1.0)	1.365 (0.876, 9.857)	**12.635 (5.362, 88.528)**	**0.021**
Longer hospital stays				
N, (%)	10 (9.4)	18 (17.1)	33 (31.1)	**0.001**
Unadjusted RR (95% CI)	Ref (1.0)	1.966 (0.861, 4.489)	**4.354 (2.014, 9.413)**	**0.001**
Adjusted RR (95% CI)	Ref (1.0)	1.680 (0.694, 4.065)	**2.673 (1.149, 6.221)**	0.056

RR, Relative risk.

Models were adjusted for age, sex, hypertension, diabetes mellitus, coronary heart diseases, other chronic heart disease, cerebrovascular diseases, chronic liver diseases, hepatitis B, chronic kidney diseases, treatments of corticosteroids, antibiotics, antiviral, and anticoagulant. Bold values indicate statistical significance.

### 3.7 The predictive powers for critical cases and death in COVID-19 patients

**Figure 4:**
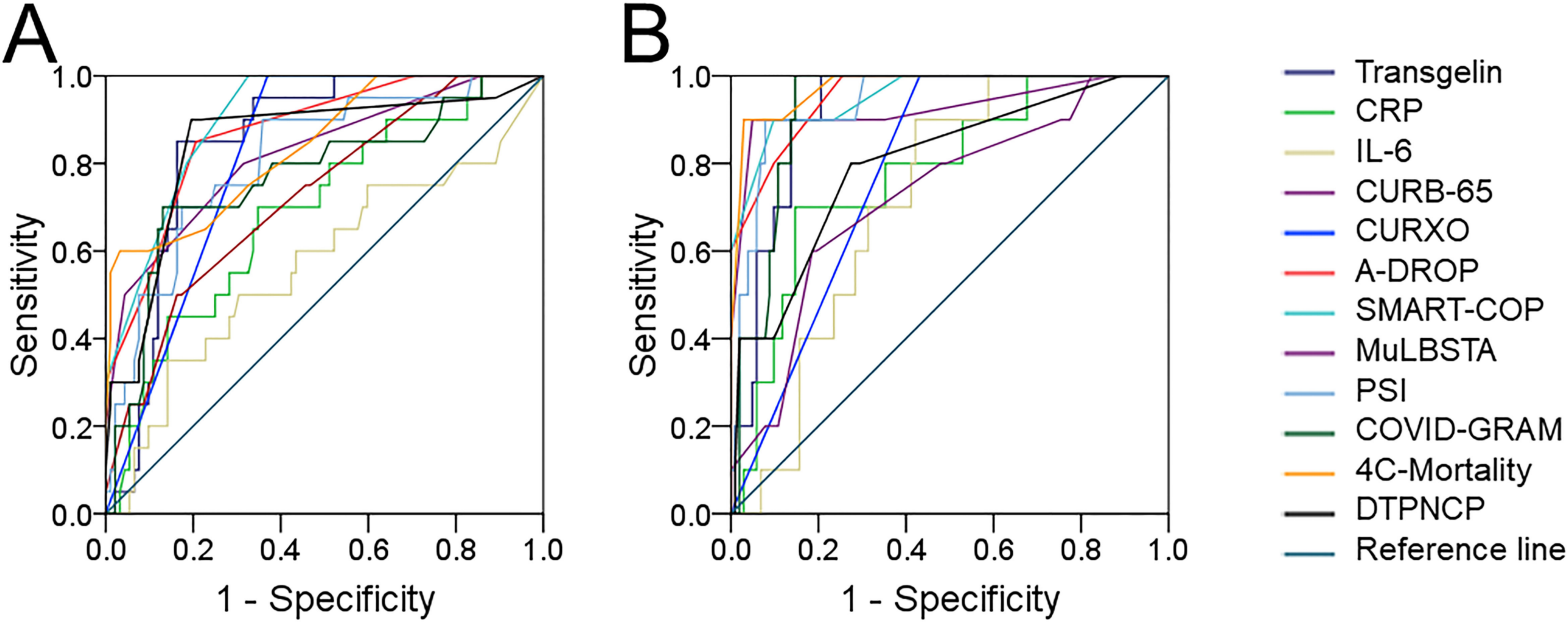
The predictive capacities for critical cases and death among COVID-19 patients. The predictive capacities for critical cases and death were assessed via ROC curve. **(A)** The predictive capacity for critical cases between serum transgelin and COVID-19 severity scores. **(B)** The predictive capacity for death between serum transgelin and COVID-19 severity scores.

The predictive powers for critical cases were assessed by ROC. As shown in **Figure 4A**, the predictive powers for critical cases were as shown below: serum transgelin: 0.769; CURB-65: 0.751; A-DROP: 0.839; SMART-COP: 0.904; MuLBSTA: 0.769; PSI: 0.758; COVID-GRAM: 0.747; 4C-Mortality: 0.798; CURXO: 0.833; DTPNCP: 0.823; IL-6: 0.699; CRP: 0.690. Serum transgelin had a cutoff value of 0.95 ng/mL to discriminate the critical patients. The sensitivity and specificity were 90.2% and 83.5, respectively. Moreover, the predive capacities for death were analyzed. As shown in **Figure 4B**, the power capacities for death were as follows: serum transgelin: 0.879; CURB-65: 0.942; A-DROP: 0.921; SMART-COP: 0.930; MuLBSTA: 0.707; PSI: 0.874; COVID-GRAM: 0.912; 4C-Mortality: 0.970; CURXO: 0.808; DTPNCP: 0.855; IL-6: 0.721; CRP: 0.778. The cutoff concentration of transgelin was 1.12 ng/mL. The sensitivity was 94.4% and the specificity was 77.1%.

## 4 Discussion

In the current investigation, serum transgelin was detected for the first time among COVID-19 patients. The data indicated that the expression of serum transgelin was ascended in line with COVID-19 severity scores. And, the expression of serum transgelin was obviously decreased in the convalescent phase after treatment. Moreover, the level of serum transgelin was strongly related to many clinical characteristics. Furthermore, serum transgelin expression on admission had a positive relationship respectively with severity scores and poorly prognostic outcomes during hospitalization. To a certain extent, the expression of serum transgelin on admission can indicate the disease condition and predict the prognosis ahead. This study found a potential role of transgelin in COVID-19 patients.

Transgelin is predominantly localized in the cytoskeleton and used as an earlier biomarker of smooth muscle differentiation (11, 12). Aberrant expression of transgelin has been depicted to be linked with many tumors related diseases (13–16). Interestingly, transgelin expression disorder is associated with a serious of pulmonary diseases. The previous investigations have elucidated that transgelin takes parts in the process of lung cancer, asthma, and lung fibrosis (17–20). In the present study, serum transgelin expression on admission was increased in parallel with COVID-19 severity scores. After treatment, serum transgelin was reduced during the convalescent phase. Correlative analyses found that serum transgelin expression was strongly related to the levels of many clinical parameters in COVID-19 patients. Further, regression analyses revealed that serum transgelin expression was positively related to the severity scores. Collectively, all of these outcomes indicated that serum transgelin expression on admission can indicate the severity of COVID-19.

Mounting evidences suggested that the expression of transgelin is associated with the prognosis of many tumors diseases. Pulmonary transgelin increase is related to tumor progression in lung adenocarcinoma (30). Additionally, transgelin overexpression leads to a poor prognosis in metastatic renal cell carcinoma (31). Moreover, transgelin is an adverse prognostic factor of tumor growth and migration in colorectal cancer patients (15). Therefore, we wanted to know the relationships of serum transgelin level with the prognostic outcomes of COVID-19 cases. In the present study, the numbers of mechanical ventilation, vasoactive agent usage, ICU admission, death, and longer hospital stays were increased with the elevated transgelin expression during hospitalization. Further analyses demonstrated that serum transgelin expression on admission was positively related to the adversely prognostic risks in COVID-19 patients. The previous study has revealed that inflammation is implicated in the process of COVID-19 (32). In addition, the predictive powers of different parameters for critical cases and death were compared among COVID-19 patients. The results revealed that the predictive capacities of serum transgelin for critical cases and death were similar with COVID-19 severity scores, and obviously higher compared with IL-6 and CRP. Overall, follow-up research provided new evidence that transgelin elevation on admission increases the risk of poor prognosis in COVID-19 cases during hospitalization.

Due to serum transgelin was only a single indicator, this meant that it was easily to detect and less time spent in the clinical practice. Not only that, the assessed efficiency of serum transglin was not less than COVID-19 severity scores and was a little higher than those in commonly inflammatory cytokines, such as IL-6 and CRP. Therefore, serum transgelin level may be used to estimate the illness state and predict the prognosis for COVID-19 patients. And it can help to make clinical decision. The current study has demonstrated that the higher serum transgelin on admission was, the higher risk of poor prognosis during hospitalization was. These results indicated that the COVID-19 patients whose the levels of serum transgelin on admission were higher than the cut-off concentrations were more likely to develop critical cases or die during hospitalization. Consequently, when the clinicians met COVID-19 patients with highly serum transgelin, we should pay more attention to these cases and avoided the disease progression and death during hospitalization. Therefore, we could consider to initiate institute early therapeutic interventions, including respiratory support, the usage of vasoactive agent, and ICU admission, to improve the evolution or even stop the progression from moderate to severe forms, and to reduce the risk of death. Although this study has hinted that serum transgelin expression may be regarded as a biomarker for monitoring the severity and prognosis among CVOID-19 patients. However, this research was only an observational and epidemiological investigation. The specific role of transgelin in the progression of COVID-19 was not explored in this research. Transgelin knockout should be executed, and the effect of transgelin knockout on the process was needed to conduct in mice model of COVID-19. Only intervention experiment can better reveal the role and significance of transgelin in COVID-19.

Assessing the relationships of serum transgelin expression with severity and outcomes are beneficial to illuminate the role of transgelin in COVID-19 patients. However, there were some flaws in this epidemiological study. First, this was a relative sample size, a larger sample size was needed to further confirm the above results. Second, this was just epidemiological research, the mechanism of transgelin elevation can’t be explored in COVID-19 cases. More *in vitro* and vivo experiments should be conducted in the following study. Third, transgelin was only measured in serum samples, the expression of transgelin wasn’t detected in both lung specimens and alveolar lavage fluid.

## 5 Conclusion

To sum up, this study primarily found that serum transgelin expression on admission was positively related to the severity and poor prognosis in COVID-19 cases. The evidences above revealed that transgelin may involve in the progression of COVID-19, supporting its probable usefulness as a serum biomarker for estimating illness state and prognosis of COVID-19 patients. The development and utilization of transgelin may assist in managing COVID-19 cases on the basis of disease extent.

## Data Availability

The raw data supporting the conclusions of this article will be made available by the authors, without undue reservation.

## References

[1] Li Q Guan X Wu P Wang X Zhou L Tong Y. Early transmission dynamics in wuhan, China, of novel coronavirus-infected pneumonia. N Engl J Med. (2020) 382:1199–207. doi: 10.1056/NEJMoa2001316 PMC7121484 31995857

[2] Wang D Hu B Hu C Zhu F Liu X Zhang J. Clinical characteristics of 138 hospitalized patients with 2019 novel coronavirus-infected pneumonia in wuhan, China. JAMA. (2020) 323:1061–9. doi: 10.1001/jama.2020.1585 PMC7042881 32031570

[3] Zhou F Yu T Du R Fan G Liu Y Liu Z. Clinical course and risk factors for mortality of adult inpatients with COVID-19 in Wuhan, China: a retrospective cohort study. Lancet. (2020) 395:1054–62. doi: 10.1016/S0140-6736(20)30566-3 PMC7270627 32171076

[4] Gavriatopoulou M Ntanasis-Stathopoulos I Korompoki E Fotiou D Migkou M Tzanninis IG. Emerging treatment strategies for COVID-19 infection. Clin Exp Med. (2021) 21:167–79. doi: 10.1007/s10238-020-00671-y PMC7598940 33128197

[5] Berlin DA Gulick RM Martinez FJ. Severe covid-19. N Engl J Med. (2020) 383:2451–60. doi: 10.1056/NEJMcp2009575 32412710

[6] Fu L Li XY Fei J Xiang Y Xiang HX Li MD. Myocardial injury at early stage and its association with the risk of death in COVID-19 patients: A hospital-based retrospective cohort study. Front Cardiovasc Med. (2020) 7:590688. doi: 10.3389/fcvm.2020.590688 33195480 PMC7661636

[7] Fu L Fei J Xu S Xiang HX Xiang Y Hu B. Liver dysfunction and its association with the risk of death in COVID-19 patients: A prospective cohort study. J Clin Transl Hepatol. (2020) 8:246–54. doi: 10.14218/JCTH.2020.00043 PMC7562804 33083246

[8] Gandhi RT Lynch JB Del Rio C. Mild or moderate covid-19. N Engl J Med. (2020) 383:1757–66. doi: 10.1056/NEJMcp2009249 32329974

[9] Karim SSA Karim QA. Omicron SARS-CoV-2 variant: a new chapter in the COVID-19 pandemic. Lancet. (2021) 398:2126–8. doi: 10.1016/S0140-6736(21)02758-6 PMC8640673 34871545

[10] Lees-Miller JP Heeley DH Smillie LB. An abundant and novel protein of 22 kDa (SM22) is widely distributed in smooth muscles. Purification from bovine aorta. Biochem J. (1987) 244:705–9. doi: 10.1042/bj2440705 PMC1148053 3446186

[11] Winder SJ Jess T Ayscough KR. SCP1 encodes an actin-bundling protein in yeast. Biochem J. (2003) 375:287–95. doi: 10.1042/BJ20030796 PMC1223692 12868959

[12] Xu R Ho YS Ritchie RP Li L. Human SM22 alpha BAC encompasses regulatory sequences for expression in vascular and visceral smooth muscles at fetal and adult stages. Am J Physiol Heart Circ Physiol. (2003) 284:H1398–407. doi: 10.1152/ajpheart.00737.2002 12521938

[13] Tsui KH Lin YH Chang KS Hou CP Chen PJ Feng TH. Transgelin, a p53 and PTEN-upregulated gene, inhibits the cell proliferation and invasion of human bladder carcinoma cells in *vitro* and in *vivo* . Int J Mol Sci. (2019) 20:4946. doi: 10.3390/ijms20194946 31591355 PMC6801752

[14] Wen F Sun X Sun C Dong Z Jia G Bao W. TAGLN is downregulated by TRAF6-mediated proteasomal degradation in prostate cancer cells. Mol Cancer Res. (2021) 19:1113–22. doi: 10.1158/1541-7786.MCR-20-0513 33771884

[15] Elsafadi M Manikandan M Almalki S Mahmood A Shinwari T Vishnubalaji R. Transgelin is a poor prognostic factor associated with advanced colorectal cancer (CRC) stage promoting tumor growth and migration in a TGFβ-dependent manner. Cell Death Dis. (2020) 11:341. doi: 10.1038/s41419-020-2529-6 32393769 PMC7214449

[16] Wei X Lou H Zhou D Jia Y Li H Huang Q. TAGLN mediated stiffness-regulated ovarian cancer progression via RhoA/ROCK pathway. J Exp Clin Cancer Res. (2021) 40:292. doi: 10.1186/s13046-021-02091-6 34538264 PMC8451140

[17] Sun C Zhang K Ni C Wan J Duan X Lou X. Transgelin promotes lung cancer progression via activation of cancer-associated fibroblasts with enhanced IL-6 release. Oncogenesis. (2023) 12:18. doi: 10.1038/s41389-023-00463-5 36990991 PMC10060230

[18] Fu J Wang X Yue Q. Functional loss of TAGLN inhibits tumor growth and increases chemosensitivity of non-small cell lung cancer. Biochem Biophys Res Commun. (2020) 529:1086–93. doi: 10.1016/j.bbrc.2020.06.066 32819569

[19] Yin LM Xu YD Peng LL Duan TT Liu JY Xu Z. Transgelin-2 as a therapeutic target for asthmatic pulmonary resistance. Sci Transl Med. (2018) 10:eaam8604. doi: 10.1126/scitranslmed.aam8604 29437149 PMC6310021

[20] Yu H Königshoff M Jayachandran A Handley D Seeger W Kaminski N. Transgelin is a direct target of TGF-beta/Smad3-dependent epithelial cell migration in lung fibrosis. FASEB J. (2008) 22:1778–89. doi: 10.1096/fj.07-083857 18245174

[21] Wang JL Chen X Xu Y Chen YX Wang J Liu YL. The associations of serum IL-37 with the severity and prognosis in patients with community-acquired pneumonia: A retrospective cohort study. Front Immunol. (2021) 12:636896. doi: 10.3389/fimmu.2021.636896 34025645 PMC8138168

[22] Hua DX Ma KS Cheng JY Liu Y Sun J He QY. Serum TRAIL predicts severity and prognosis in patients with community-acquired pneumonia: a prospective cohort study. Intern Emerg Med. (2022) 17:2279–90. doi: 10.1007/s11739-022-03086-7 PMC9569003 36241932

[23] Liang W Liang H Ou L Chen B Chen A Li C. Development and validation of a clinical risk score to predict the occurrence of critical illness in hospitalized patients with COVID-19. JAMA Intern Med. (2020) 180:1081–9. doi: 10.1001/jamainternmed.2020.2033 PMC7218676 32396163

[24] Gordon AJ Govindarajan P Bennett CL Matheson L Kohn MA Camargo C. External validation of the 4C Mortality Score for hospitalised patients with COVID-19 in the RECOVER network. BMJ Open. (2022) 12:e054700. doi: 10.1136/bmjopen-2021-054700 PMC9023850 35450898

[25] Jin Z Liu JY Feng R Ji L Jin ZL Li HB. Drug treatment of coronavirus disease 2019 (COVID-19) in China. Eur J Pharmacol. (2020) 883:173326. doi: 10.1016/j.ejphar.2020.173326 32598953 PMC7319927

[26] Iijima Y Okamoto T Shirai T Mitsumura T Sakakibara R Honda T. MuLBSTA score is a useful tool for predicting COVID-19 disease behavior. J Infect Chemother. (2021) 27:284–90. doi: 10.1016/j.jiac.2020.10.013 PMC7552979 33129694

[27] Ahn JH Choi EY. Expanded A-DROP score: A new scoring system for the prediction of mortality in hospitalized patients with community-acquired pneumonia. Sci Rep. (2018) 8:14588. doi: 10.1038/s41598-018-32750-2 30275523 PMC6167349

[28] Li W Zhao X Yu TT Hao W Wang GG. Knockout of PKC θ gene attenuates oleic acid-induced acute lung injury via reduction of inflammation and oxidative stress. Iran J Basic Med Sci. (2021) 24:986–91. doi: 10.22038/ijbms.2021.56908.12695 PMC8528254 34712430

[29] Pu Z Wang W Xie H Wang W. Apolipoprotein C3 (ApoC3) facilitates NLRP3 mediated pyroptosis of macrophages through mitochondrial damage by accelerating of the interaction between SCIMP and SYK pathway in acute lung injury. Int Immunopharmacol. (2024) 128:111537. doi: 10.1016/j.intimp.2024.111537 38232538

[30] Wu X Dong L Zhang R Ying K Shen H. Transgelin overexpression in lung adenocarcinoma is associated with tumor progression. Int J Mol Med. (2014) 34:585–91. doi: 10.3892/ijmm.2014.1805 24938684

[31] Bouchalova P Beranek J Lapcik P Potesil D Podhorec J Poprach A. Transgelin contributes to a poor response of metastatic renal cell carcinoma to sunitinib treatment. Biomedicines. (2021) 9:1145. doi: 10.3390/biomedicines9091145 34572331 PMC8467952

[32] Pu Z Sui B Wang X Wang W Li L Xie H. The effects and mechanisms of the anti-COVID-19 traditional Chinese medicine, Dehydroandrographolide from Andrographis paniculata (Burm.f.) Wall, on acute lung injury by the inhibition of NLRP3-mediated pyroptosis. Phytomedicine. (2023) 114:154753. doi: 10.1016/j.phymed.2023.154753 37084628 PMC10060206

